# Effects of high-flow nasal cannula therapy on oxygenation, lung volumes and CO_2_ removal in critically ill hypoxemic patients: preliminary results

**DOI:** 10.1186/cc14281

**Published:** 2015-03-16

**Authors:** T Mauri, N Eronia, G Bellani, G Grasselli, R Marcolin, S Marocco Arrigoni, A Pesenti

**Affiliations:** 1 IRCC Ca' Granda Maggiore Policlinico Hospital, Milan, Italy; 2 San Gerardo Hospital, Monza, Italy

## Introduction

High-flow nasal cannula (HFNC) is increasingly proposed as respiratory support for hypoxemic non-intubated acute respiratory failure patients. Clinically, HFNC therapy decreases dyspnea, improves patient's comfort, improves oxygenation and enhances clearance of upper airway secretions [1]. We present preliminary results from a clinical study aimed at measuring the effects of HFNC on gas exchange, lung volumes and inspiratory effort in hypoxemic non-intubated critically ill patients.

## Methods

We performed a prospective randomized cross-over study on hypoxemic non-intubated patients (PaO_2_/FiO_2_ ≤300 mmHg) admitted to the ICU of the San Gerardo Hospital and prescribed to receive oxygen by facial mask. We delivered the same air/oxygen mix by HFNC (Optiflow; Fisher & Paykel Healthcare, Auckland, New Zealand) and facial mask (20 minutes per step). Continuous recordings of regional lung volumes by EIT (Pulmovista 500; Drager Medical GmbH, Lubeck, Germany) and of inspiratory effort by esophageal pressure (Pes) were obtained and analyzed offline by dedicated software.

## Results

We enrolled 15 patients (10 male), age 57 ± 16 years. Compared with standard facial mask, HFNC significantly improved PaO_2_/FiO_2 _(199 ± 60 vs. 150 ± 46, *P *< 0.001) and end-expiratory lung impedance (corresponding to aeration) (866 ± 568 au vs. baseline, *P *< 0.001). Moreover, HFNC decreased the respiratory rate (22 ± 5 bpm vs. 20 ± 5 bpm, *P *< 0.001), as well as negative Pes swings (ΔPes 8.3 ± 5 mmHg vs. 6.6 ± 1 mmHg, *P *< 0.01) and corrected minute ventilation (that is, actual MV × actual PaCO_2_ / 40 mmHg) (49,887 ± 16,176 au vs. 41,811 ± 14,042 au, *P *< 0.001). Finally, central venous pressure increased (6 ± 5 mmHg vs. 4 ± 5 mmHg, *P *< 0.01), possibly indicating positive end-expiratory pressure effect.

## Conclusion

In non-intubated hypoxemic critically ill patients, HFNC improves oxygenation and end-expiratory aeration; moreover, HFNC reduces the inspiratory effort and the minute ventilation needed to maintain normal arterial CO_2_ tension.
